# Sleep Recovery Restored Neuroglobin Immunoreactivity in Rat LDTg-PPTg Nuclei

**DOI:** 10.1155/2020/8353854

**Published:** 2020-07-22

**Authors:** Montserrat Melgarejo-Gutiérrez, Fabio García-García, Gerardo Hernández-Márquez, Consuelo Morgado-Valle, Mario Eduardo Acosta-Hernández, Juan Carlos Rodríguez-Alba

**Affiliations:** ^1^School of Medicine, Veracruzana University, Xalapa, Veracruz 91055, Mexico; ^2^Biomedicine Department, Health Science Institute, Veracruzana University, Xalapa, Veracruz 91190, Mexico; ^3^Brain Research Center, Veracruzana University, Xalapa, Veracruz 91055, Mexico

## Abstract

Neuroglobin (Ngb) is a protein member of the globin family, expressed mainly in the central and peripheral nervous system. It is involved in the transport of oxygen in response to hypoxic/ischemic and oxidative stress-related insults. We recently showed that sleep deprivation reduces the number of Ngb-positive cells in brain areas related to sleep. However, it is poorly understood whether Ngb expression correlates with sleep occurrence. Here, we aimed to study if sleep recovery produced by 24 h of sleep deprivation restores the number of Ngb-positive cells in the pedunculopontine tegmentum (PPTg) and laterodorsal tegmentum (LDTg), brain areas related to sleep-wake regulation. Male Wistar rats were sleep-deprived for 24 h using the gentle handling method. After sleep deprivation, rats were allowed a sleep recovery for three or six hours. After sleep recovery, rats were euthanized, and their brains processed for Ngb immunohistochemistry. We found that a 3 h sleep recovery is enough to restore the number of Ngb-positive cells in all the analyzed areas. A similar result was observed after a 6 h sleep recovery. These results suggest that Ngb expression is sleep dependent. We suggest that Ngb expression is involved in preventing cell damage due to prolonged wakefulness.

## 1. Introduction

Neuroglobin (Ngb) is a monomeric globin with a high affinity for oxygen and located mainly in the neurons [1]. Ngb acts as an oxygen reservoir and is a scavenger of reactive oxygen species and nitric oxide [2]. It is reported that Ngb expression increases after neuronal hypoxia in vitro and after focal cerebral ischemia in vivo [3, 4]. The size of cerebral infarction after middle cerebral artery occlusion is reduced by 30% in transgenic mice overexpressing Ngb [5]. Ngb overexpression is also protective against Alzheimer's disease [6]. All these pieces of evidence suggest that Ngb has a protective role when low oxygen levels are present.

Interestingly, the pedunculopontine tegmentum (PPTg) and laterodorsal tegmentum (LDTg) nuclei have a high Ngb immunoreactivity [7]. PPTg and LDTg neurons are necessary for rapid eye movement (REM) sleep to occur; the lesion of these nuclei decreases REM sleep [8]. Both nuclei increase their firing activity during REM sleep [9]. Besides, the number of c-fos-positive cells increases in both nuclei under conditions that increase REM sleep duration [10, 11]. We have previously described that 24 h sleep deprivation in rats reduces the number of Ngb-positive cells in PPTg and LTDg, suggesting that sleep is necessary to Ngb expression in these nuclei. The reduction of Ngb-positive cells was independent of the physiological stress induced by sleep deprivation since corticosterone serum levels have no change in rats after manipulation. Furthermore, oxidative stress measured by lipid peroxidation was not different between the brain areas from control and sleep-deprived rats [12]. In this sense, sleep onset is necessary for Ngb expression.

A study showed that 117 proteins, a total of 309 detected in serum samples from chronic sleep-deprived rats, showed more than 1.8-fold abundance alterations between nondeprived and sleep-deprived rats [13]. Besides, clinical and experimental evidence suggests that sleep deprivation may increase the risk of impaired cognition, cardiovascular disorders, and metabolic alterations. Several of these disorders may be due to changes in protein expression, potentially facilitated by reduced hours of sleep. It is well documented that forced sleep deprivation in rats is followed by compensatory increases in sleep duration and sleep intensity, measured by slow-wave activity (SWA) during nonrapid eye movement (NREM sleep), known as sleep rebound [14, 15].

Sleep provides a restorative function to the body to recover from prior wakefulness and fatigue by repairing processes and restoring energy. We hypothesize that Ngb expression is sleep-dependant, and for that reason, sleep recovery produced by sleep deprivation restores the number of Ngb-positive cells. Hence, the objective of the present study was to determine whether sleep restores the number of Ngb-positive cells. Our results show that the number of Ngb-positive cells into the PPTg and LDTg is restored after sleep recovery produced by sleep deprivation.

## 2. Materials and Methods

### 2.1. Animals

Male Wistar rats (250-300 g) were housed at 22°C ± 1°C in a 12 : 12 light-to-dark cycle (lights on at 09:00), with food and water ad libitum. All experimental procedures were approved and conducted according to the Institutional Ethical Committee (CICUAL-2015-0014), in agreement with the national (NOM-062-ZOO-1999) guidelines; also, we followed the US National Institutes of Health (NIH) guidelines for animal care and handling. All precautions were taken to minimize the pain or discomfort of the animals and to minimize the number of animals used.

### 2.2. Surgery and Implantation of Sleep Electrodes

After deep anesthesia with ketamine-xylazine (87 and 13 mg/kg, respectively), three stainless steel miniature screws, used as electrodes, were implanted over the frontal and parietal bones to record a cortical electroencephalogram (EEG) and Teflon-coated wires placed bilaterally into both trapezius muscles to record electromyographic (EMG) activity. Cortical and muscular electrodes were soldered to a recording plug attached to the skull with dental acrylic. After surgery, animals received an intramuscular injection of ketorolac and enroxil (3.5 mg/kg and 0.5 mL/kg, respectively) to reduce pain, inflammation, and prevent any infection. All animals were allowed a 7-day recovery after surgery, followed by two days of habituation into the recording chamber.

### 2.3. Sleep Recording

After habituation, EEG and EMG signals were recorded during 24 h with the Sirenia© sleep recording system, starting at 09:00 AM. Sleep-wake cycle stages were manually scored in epochs using standard criteria as follows: wakefulness: EEG with low-amplitude and high-frequency waves coupled to high-voltage EMG activity; NREM sleep: EEG rich in high-amplitude and low-frequency waves and low voltage for EMG; and REM sleep: highly regular theta EEG activity and loss of muscle tone with occasional twitches. At least 80% of each stage per epoch was considered an episode of the stage.

### 2.4. Sleep Deprivation and Sleep Recovery

**Figure 1:**
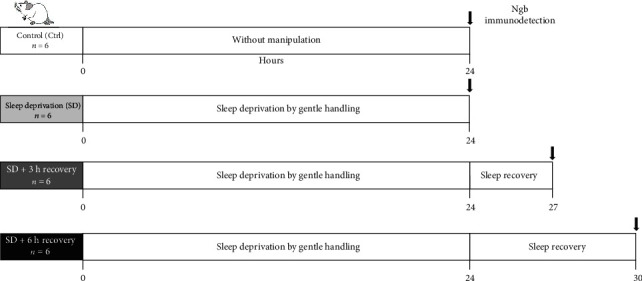
Experimental groups: the control group (Ctrl), rats without manipulation; sleep-deprived group (SD), rats sleep-deprived for 24 h; SD + 3 h, rats sleep-deprived for 24 h and sleep recovery for three hours; and SD + 6 h sleep recovery, rats sleep-deprived for 24 h and sleep recovery for six hours. After manipulation, rats were perfused, and their brains processed to Ngb immunodetection.

All rats were habituated to the researchers one week before starting the experiment. In the control group (*n* = 6), the rats were maintained for 24 h in their cages, and sleep recording was performed. Three groups of rats (*n* = 6 for each group) were sleep-deprived during 24 h, starting at 09:00 AM (lights-on) using the gentle handling method [16]. EEG and EMG activities were recorded during all times of SD, assuring that the animals did not show bouts of microsleep, which are characteristic of prolonged sleep loss. A rotatory shift was scheduled to ensure that a particular researcher would carry out the sleep deprivation for 3 h periods at a time (8 shifts/24 h). After sleep deprivation (SD), a group of rats was euthanized immediately (*n* = 6). The other groups of rats were allowed to sleep for three or six hours, respectively. The 12 : 12 h photoperiod was preserved during the 24 h of sleep deprivation and recovery. During the lights-off period, a dim red light was turned on to continue with the sleep deprivation. After sleep recovery, the rats were euthanized, and their brains processed for Ngb immunohistochemistry (Figure 1).

### 2.5. Neuroglobin Immunohistochemistry

The rats from all experiments were deeply anesthetized with sodium pentobarbital i.p. and transcardially perfused with saline solution (0.9%), followed by paraformaldehyde (4%) in 0.1 M phosphate buffer (PB) pH 7.4. The brains were removed and maintained in the fixative solution overnight and then equilibrated to a gradient of sucrose solution (10, 20, and 30%). Coronal sections (40 *μ*m thick) from the PPTg (Bregma −8.16 to −8.64), and LDTg (Bregma −8.16 to −8.40) according to Paxinos [17] were obtained using a cryostat (Hyrax C25 Microm, Zeiss). The tissue was washed in PB four times and then exposed 10 min to 1% hydrogen peroxide to neutralize endogenous peroxidase. The sections were washed four times with PB and then incubated for 48 hours at 4°C with the anti-Ngb polyclonal rabbit antibody (sc-30144; Santa Cruz Biotechnology Santa Cruz, CA), diluted 1 : 2000 in PB+3% normal goat serum with 0.3% Triton X-100 (T-9284 Sigma, St. Louis, MO, USA). The sections were washed four times with PB and incubated for 2 hours with a biotinylated goat anti-rabbit antibody (656140, Invitrogen Laboratories USA), diluted 1 : 500 in 3% normal goat serum with 0.3% Triton X-100. After four washes of PB, slices were incubated in the avidin-biotin-HRP complex (1 : 250, Pk-6100 Elite Kit, Vector Laboratories, Burlingame, CA) for 1 hour. The peroxidase activity was visualized by reaction with a solution of 0.05% diaminobenzidine (D-8001, Sigma, St. Louis, MO, USA) in the presence of nickel sulfate (1%), cobalt chloride solution (1%) (Sigma A1827 and 202185, respectively), and 0.01% hydrogen peroxide. The sections were mounted on gelatin-coated slides, dehydrated, and cleared in xylene, then coverslipped with Permount. The control sections were processed as above but with the primary antibody omitted.

### 2.6. Quantification of Ngb+ Cells

Ngb immunoreactivity was identified as a black-purple precipitate from the DAB-nickel/cobalt reaction in the cell perikarya. All slides were coded. The number of Ngb+ nuclei was counted bilaterally in both hemispheres by two observers blind to the experimental condition, using a rectangular grid in a Nikon microscope (Eclipse E200) with a digital camera (Dxm 1200C). Six sections from PPTg and LDTg per animal were analyzed to count only darkly stained cells; a gray threshold level for Ngb+ labeling was set each image using ImageJ software (ImageJ 1.48; NIH, Bethesda, MD). Cell counts for each rat were summed across all sections, and the average number of cells was calculated according to previous reports [12, 16].

### 2.7. Statistics Analysis

All data are presented as mean ± SEM, one-way analysis of variance was used for comparisons between groups. Multiple comparisons versus the control group were made according to Dunn's method. Statistical significance was established at *p* < 0.05.

## 3. Results and Discussion

### 3.1. Results

#### 3.1.1. Sleep Recovery Restores the Number of Ngb+ Cells

Sleep deprivation by 24 h reduces the total time spent in sleep. The percentage of wakefulness was significantly increased in sleep-deprived rats. NREM sleep also was reduced (*p* < 0.01). REM sleep was abolished during all sleep recordings (Table 1).

Data are percentages of time of sleep in different sleep-wake stages. Sleep deprivation: SD group. Em dash (—) indicates that REM sleep was not observed. Data are expressed as means ± S.E.M.∗*p* < 0.01.

**Figure 2:**
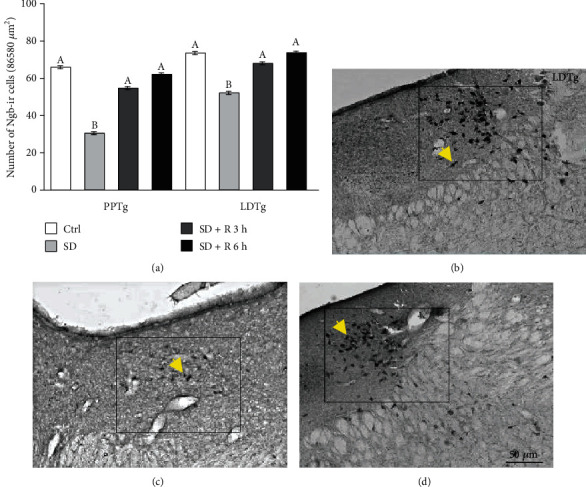
The sleep-restored neuroglobin (Ngb) immunoreactivity. (a) The total number of Ngb-positive cells after three and six hours of sleep recovery. Sleep deprivation (SD) reduces the Ngb-positive cells in all nuclei analyzed compared to control (Ctrl) ((a) vs. (b)). No significant difference was observed between the Ctrl group versus three and six hours of sleep recovery. Significant differences were found between sleep recovery groups vs. SD. Pedunculopontine tegmentum (PPTg) and laterodorsal tegmentum (LDTg) nuclei. The next panel is representative photomicrographs of LDTg sections from Ctrl (b), SD (c), and SD + 3 h of sleep recovery (d) groups, respectively. Arrow indicates Ngb-positive cells. Data are expressed as the mean±SEM. Magnification 20x. The rectangle (333 × 260 *μ*m) represents the area that was counted. Scale bar: 50 *μ*m. *b* = *p* < 0.05.

Concerning the number of Ngb-positive cells, sleep deprivation reduced the Ngb immunoreactive cells in both PPTg and LDTg nuclei (Figure 2). Interestingly, 3 h after sleep deprivation, the number of Ngb-positive cells has recovered. No differences were observed in the number of Ngb-positive cells in the LDTg nucleus between the control group and SD plus 3 h of recovery (Table 2). A similar result was observed in the PPTg (Table 2).

Values are significant: ∗*p* < 0.05 vs. control. *n* = 6 per group.

The same effect was observed in the number of Ngb-positive cells after 6 h of recovery. In the PPTg nucleus, there were no differences between the control and SD plus 6 h of recovery (control, 66.08 ± 2.78 cells; SD + recovery 6 h, 62.18 ± 3.55 cells (*n* = 6), *p* = 0.631) and in the LDTg nucleus (control, 73.19 ± 2.28 cells; SD + recovery 6 h, 73.51 ± 4.01 cells (*n* = 6), *p* = 0.717, Figure 2).

## 4. Discussion

The results corroborate that sleep deprivation by 24 h reduces the number of Ngb-positive cells and demonstrate that 3 h and 6 h of sleep recovery after 24 h of sleep deprivation were sufficient time to restore the number of Ngb-positive cells in the PPTg and LDTg nuclei. In a previous study, we demonstrated that gently handling sleep deprivation reduces the number of Ngb-positive cells significantly in these nuclei [12]. Now, our new finding suggests that sleep recovery after sleep deprivation promotes Ngb expression.

Although the rebound of electrophysiological sleep was not recorded in this study, it is well documented that during the initial 6 h of sleep recovery, the total time spent in NREM and REM is increased. Particularly, REM sleep time is increased almost twice after 24 h of sleep deprivation. Moreover, it is reported that EEG slow-wave activity (SWA; mean power density 0.75-4.0 Hz) during NREM sleep is elevated relative to a nonsleep-deprived condition, and the number of brief awakenings is reduced [14, 15]. Therefore, it is crucial to characterize whether Ngb expression is NREM or REM dependent. We can hypothesize that REM sleep could be responsible for inducing Ngb expression into neurons due to the association between PPTg and LDTg with this sleep stage. A preliminary study showed that the number of Ngb-positive cells is significantly increased at the end of the sleep period (ZT 12). In contrast, the number of positive cells at the start of the light period (ZT 0, start of sleep period) is reduced (unpublished data). Probably during sleep, Ngb levels increase progressively to reach a maximum at the beginning of wakefulness.

Ngb is highly expressed in neurons of the suprachiasmatic nucleus (SCN) in the hypothalamus that serves as the master clock of the brain mammals. SCN neurons coexpress Ngb and PER-1, a clock gene involved in the circadian regulation and maintenance of SCN activity [7]. SCN receives a monosynaptic input from the retina, where Ngb is highly expressed. Interestingly, Ngb mRNA expression in SCN neurons of mice increases during the light period, meanwhile during the dark period decreases in a clear circadian pattern [18], and neuroglobin-deficient mice disrupt the SCN light response and increase the expression of PER1 after light stimuli during the dark period [18]. It also correlated with our laboratory's preliminary results that show an increase in the number of Ngb-positive cells during sleep (light phase) and a decrease during the wake phase (dark phase). This evidence suggests that the deficiency of Ngb does not affect the circadian clock function. However, its response to the light additionally suggests that Ngb is circadian regulated but also participates in light responses. Since sleep is regulated in a circadian phase directly from SCN, it could be one of the main (onset) signals for Ngb synthesis and regulation.

The specific Ngb expression into PPTg and LDTg neurons could be related to REM sleep regulation. The PPT and LDT are made up of heterogeneous populations of cells, including distinct populations of cholinergic, GABAergic, and glutamatergic neurons [19]. In particular, GABAergic and glutamatergic neurons are active during REM sleep [20]. It is necessary to determine which neurons are expressed Ngb and whether REM sleep is directly related. Another possible way for Ngb regulation and expression is the orexinergic pathway; it has been shown that prolonged wakefulness induces an increase in orexin levels in the cerebrospinal fluid [21]. Orexins participate in the maintenance of wakefulness [22], and interestingly, Ngb-positive neurons in PPTg and LDTg coexpress the orexin-1 receptor [23].

On the other hand, in vitro studies suggest that cytosolic Ngb could react with mitochondrial cytochrome c, interfering with apoptotic pathways [24]. Prolonged wakefulness induces oxidative stress and eventually leads to cellular damage [25, 26]. This cellular damage is related to the development of neurodegenerative diseases, like Alzheimer's. Studies in double transgenic mice demonstrated that Ngb levels reduce amyloid-beta (A*β*) deposits, decrease level of A*β* (1-40) and A*β* (1-42), and improve behavioral performance, thereby abating the Alzheimer's disease phenotype [5, 27]. Therefore, Ngb could participate as an endogenous neuroprotector in situations of sleep loss and prolonged shift work. For example, patients that suffer obstructive sleep apnea report higher serum levels of Ngb, suggesting that Ngb has a protective role at low oxygen levels [28].

## 5. Conclusion

The results of the present study suggest that sleep recovery restore Ngb levels in the brain regions related to REM sleep regulation. Thus, sleep is necessary to induce Ngb expression.

## Figures and Tables

**Table 1:** Sleep values during sleep deprivation.

Stage	Control	SD 24 h	SD + 3 h	SD + 6 h
Wake	46.68 ± 2.17	98.92 ± 1.02∗	98.12 ± 1.01∗	98.75 ± 1.13∗
NREM sleep	37.83 ± 1.34	1.60 ± 0.25∗	1.88 ± 0.21∗	1.25 ± 0.25∗
REM sleep	15.49 ± 1.07	—	—	—

**Table 2:** Means ± SEM of number of Ngb-positive cells for each group.

Nucleus	Control	SD	SD + recovery 3 h	*p*	SD + recovery 6 h	*p*
LDTg	73.19 ± 2.28	51.85 ± 3.72∗	68.08 ± 3.71	0.774	73.51 ± 4.01	0.717
PPTg	66.08 ± 2.78	30.84 ± 2.29∗	54.51 ± 2.81	0.852	62.18 ± 3.55	0.631

## Data Availability

The data used to support the findings of this study are available from the corresponding author upon request.
